# Supplementary material for: Network Approach to Evaluate the Effect of Diet on Stroke or Myocardial Infarction Using Gaussian Graphical Model
Source: Nutrients. 2025 May 8;17(10):1605. doi: 10.3390/nu17101605 (PMC12114211; doi:10.3390/nu17101605)
Supplement: Supplementary file 1 [file nutrients-17-01605-s001.zip › GGM Supplementary Figures and Tables 20250430.pdf]

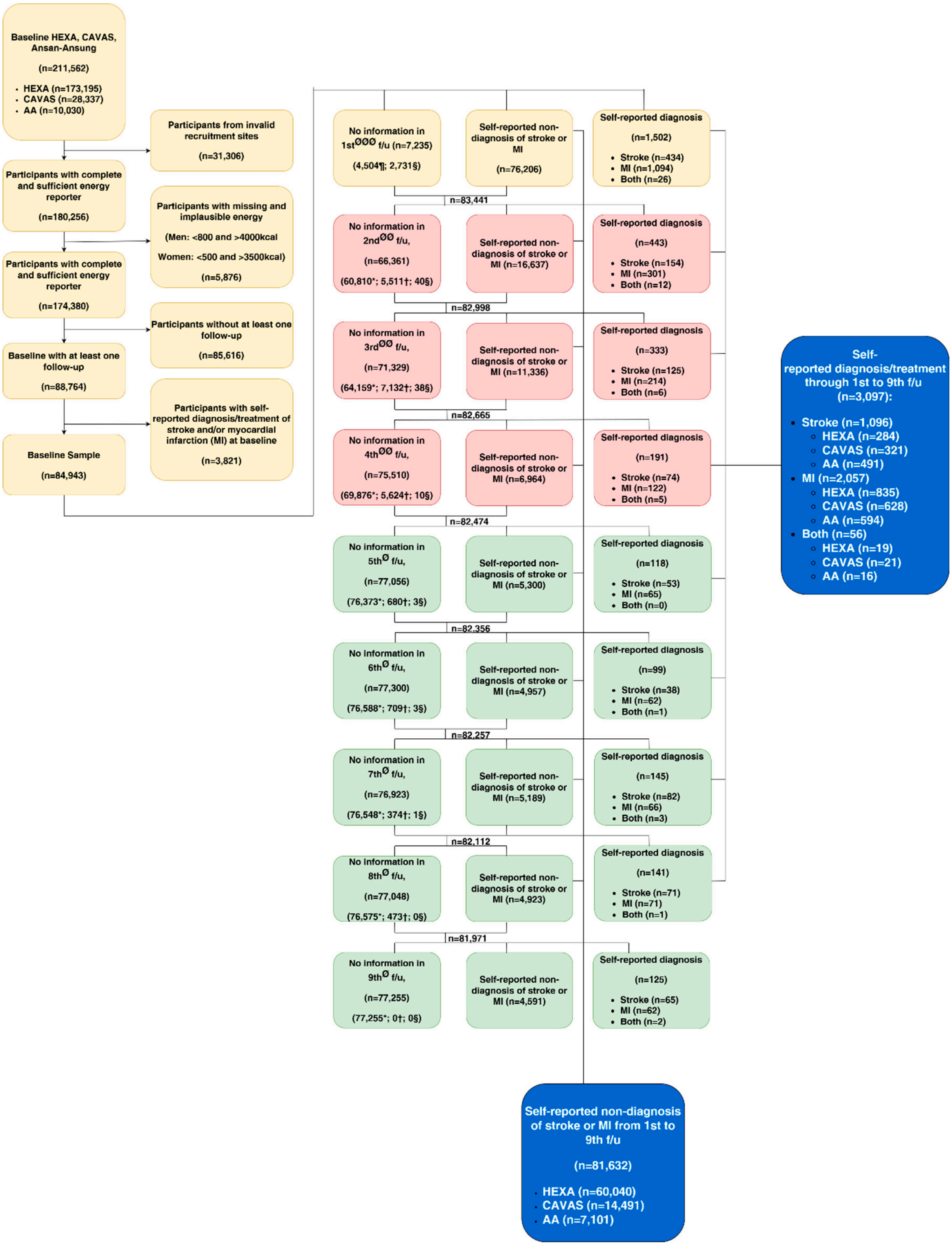

ØØØHEXA, CAVAS, Ansan-Ansung
ØØCAVAS, Ansan-Ansung
ØAnsan-Ansung

¶Those who had NOT participated in the 1st f/u
*Those who had NOT participated in the previous f/u
†Those who HAD participated in the previous f/u
§Non-reporters of self-diagnosis and treatment of Stroke or MI

Supplementary Figure S1. Selection criteria and identification of outcomes for all cohorts (detailed)

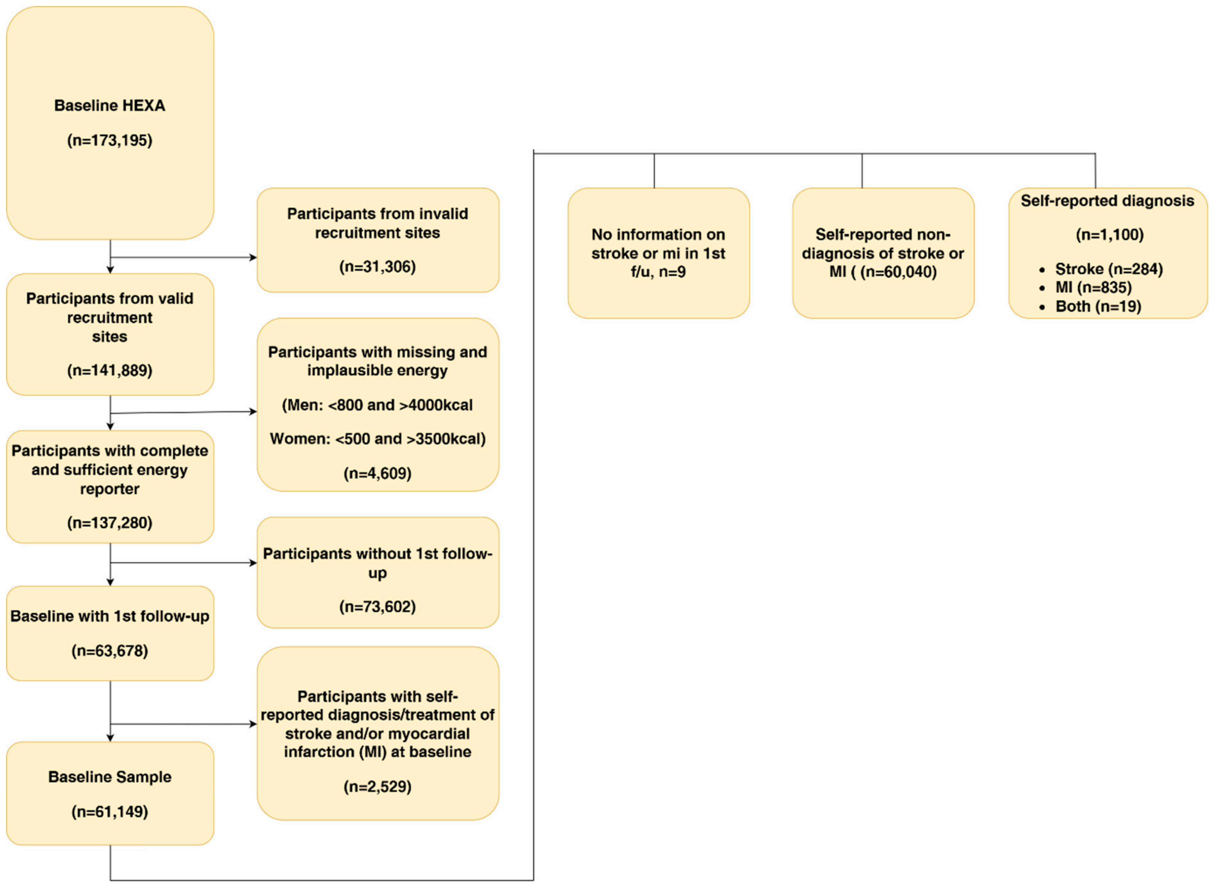

Supplementary Figure S2. Selection criteria and identification of outcomes for HEXA cohort

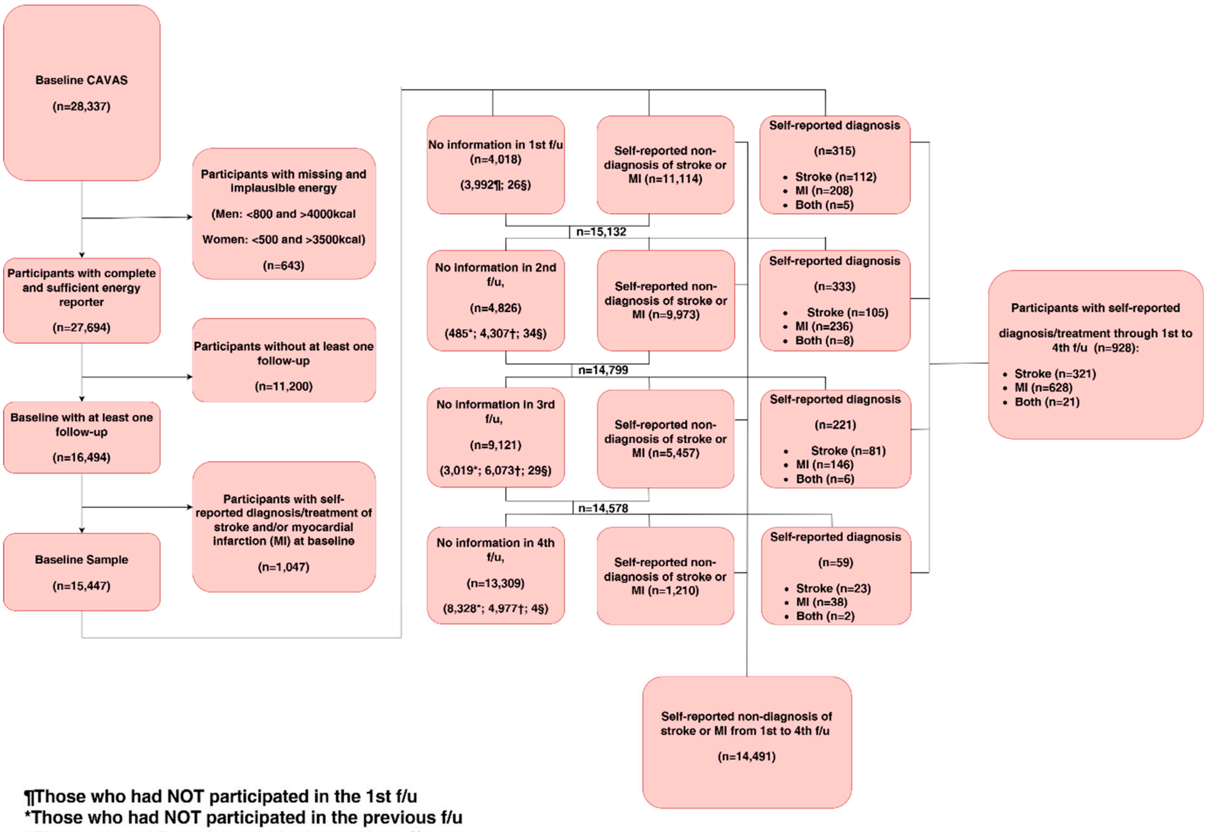

¶Those who had NOT participated in the 1st f/u
*Those who had NOT participated in the previous f/u
†Those who HAD participated in the previous f/u
§Non-reporters of self-diagnosis and treatment of Stroke or MI

Supplementary Figure S3. Selection criteria and identification of outcomes for CAVAS cohort

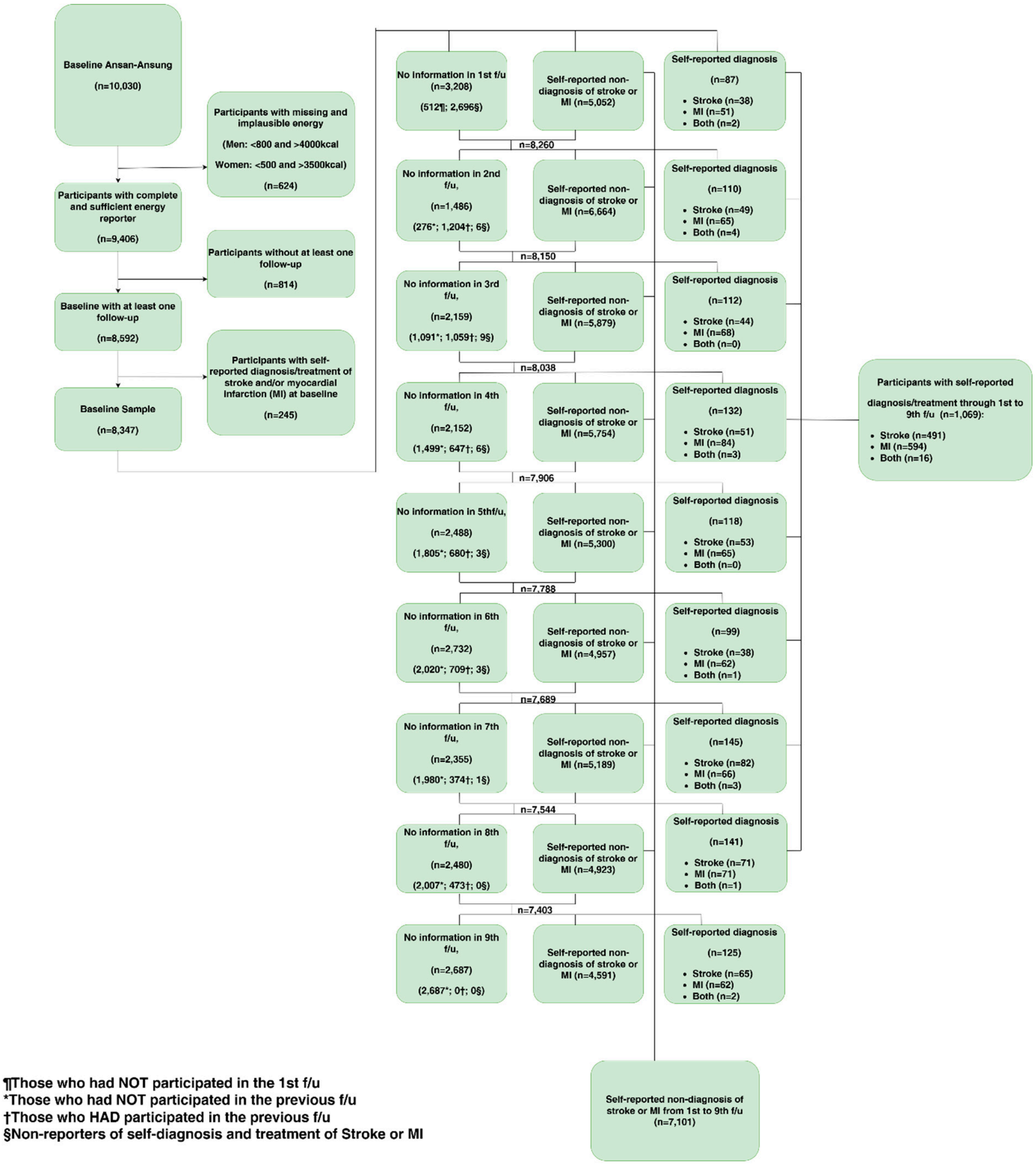

Supplementary Figure S4. Selection criteria and identification of outcomes for Ansan-Ansung cohort

Supplementary Table S1. Food group composition

Food Group	Food Item
Breads	Loaf bread / Sandwich / Toast; Bread with small red beans; Other breads; Jam/ Honey/ Margarine (when spread on bread)
Cereal	Grain powder; Cereals
Chocolate/Sweets	Cake / Chocolate pie; Cookie / Cracker / Snack; Candy / Chocolate
Dairy	Yogurt; Ice cream; Cheese
Drinks: Coffee	Coffee (without sugar); Coffee, sugar; Coffee, cream
Drinks: Green Tea	Green tea
Drinks: Others	Other drinks (Citron tea, Plum tea, Aloe, Persimmon punch, Ginseng tea, Sikhye, Jujube tea, Black herbal tea)
Drinks: Soda	Carbonated drinks (coke, sprite)
Eggs	Eggs / Quail Egg
Fish: Blue	Mackerel/Pacific saury/Spanish mackerel; Dried anchovy; Tuna, canned
Fish: White	Hair tail; Eel; Yellow croaker / Snapper / Halibut; Alaska Pollack
Fruits: Purple	Grape / Grape juice
Fruits: Red/Pink	Strawberry; Watermelon; Peach / Plum; Apple / Apple juice
Fruits: White	Korean pear / Pear juice
Fruits: Yellow/Orange	Banana; Korean melon / Melon; Persimmon; Tangerine; Orange / Orange juice
Kimchi	Kimchi, Korean cabbage; Kkakdugi / Radish kimchi; Other Kimchi (Green onion, Kodulbbagi, Mustard Leaves)
Legume	Bean
Soybean Based Food	Soybean paste soup; Tofu; Soybean milk
Meat Soup	Beef soup (Seolleongtang, Gomtang, Galbitang, Doganitang); Beef Soup with vegetables; Organ meat, Seonji, Sundae
Meat: Red/Processed	Processed meat (Ham, Sausage)
Meat: Red/Unprocessed	Pork belly; Pork, Pan roasted; Pork, braised; Steak / beef roast; Dog meat
Meat: White	Fried chicken / Whole Chicken Soup / Samgyetang / Chicken Stew
Milk	Milk
Mushroom	Oyster mushroom; Other mushroom
Noodles	Ramyon; Wheat noodles with soup (Kalguksu, Jangguk-noodles, Udon); Chajangmyon / Champpong; Naengmyeon / Buckwheat noodles; Dumpling / Dumpling soup
Nuts	Peanut / Almond / Pine nut
Pizza/Hamburger	Pizza/ Hamburger
Potatoes/starch	Starch jelly; Potatoes; Sweet potatoes; Japchae (Starch vermicelli)
Radish	Radish pickles; Nabak Kimchi / Dongchimi
Rice cake	Rice cake (plain rod shape) / Rice cake soup; Other rice cakes
Rice- Mixed	Cooked rice with beans; Cooked rice with other cereals; Half cooked well milled rice half cooked rice with beans; Half cooked well milled rice half cooked rice with other cereals; Cooked rice with barely
Rice- White	Cooked rice, well milled
Seafood: Crustacean	Crab; Shrimp
Seafood: Mollusks	Cuttlefish / Octopus; Clam / Whelk; Oysters
Seafood: Processed	Fish paste; Salt-fermented fish
Seaweed	Laver, dried; Kelp / Sea mustard
Sushi	Sliced Raw Fish
Vegetables: Allium	Onion; Crown daisies / Leek / Water dropwort
Vegetables: Brown	Bracken / Sweet potato stems / Taro stem
Vegetables: Cruciferous	Cabbage / Cabbage Soup
Vegetables: Green	Spinach; Lettuce; Perilla leaf; Other green vegetables (Shepherd's purse, Beetroot, Curled mallow, Mugwort, Outer leaves); Red pepper leaves / Chamnamul / Chwinamul; Cucumber; Green pepper
Carrot	Carrot / Carrot Juice
Vegetables: Others	Vegetable wraps / Vegetable salad; Doraji / Deoduck; Vegetable juice; Jangaji; Bean sprouts / Mung bean sprouts
Vegetables: Red	Tomato / Cherry tomato / Tomato juice
Pumpkin	Pumpkin / Sweet pumpkin / Pumpkin juice; Pumpkin, immature

Supplementary Table S2. Community composition and network centrality score

Community	Food Group	Eigenvector Centrality Score
HCPF (High Calorie and Processed Food)	Milk	1.0000
	Eggs	0.6842
	Soybean Based Food	0.6842
	Breads	0.6064
	Fish: Blue	0.4866
	Chocolate/Sweets	0.2464
	Pizza/Hamburger	0.2464
	Fish: White	0.1977
FD (Fruits and Dairy)	Fruits: Purple	1.0000
	Fruits: Red/Pink	1.0000
	Fruits: Yellow/Orange	1.0000
	Vegetables: Red	1.0000
	Dairy	0.8508
	Fruits: White	0.8508
HPGT (High Protein and Green Tea)	Meat: Red/Unprocessed	1.0000
	Drinks: Green Tea	0.6281
	Meat Soup	0.4821
	Meat: White	0.4821
	Noodles	0.4821
	Sushi	0.3028
RHCB (Rice and High Calorie Beverages)	Rice- White	1.0000
	Drinks: Others	0.7446
	Drinks: Soda	0.7446
	Rice cake	0.4268
	Rice- Mixed	0.4268
VEG (Vegetables)	Pumpkin	1.0000
	Vegetables: Others	0.9401
	Mushroom	0.7942
	Vegetables: Brown	0.7004
	Vegetables: Green	0.4970
	Carrot	0.4596
	Radish	0.3482
	Vegetables: Cruciferous	0.3300
	Potatoes/starch	0.2562
	Vegetables: Allium	0.2562
	Kimchi	0.0892

Supplementary Table S3. Median network scores per community by total population and sex

Community: Median (IQR)	Total Population (n=84,729)	Male (n=30,131)	Female (n=54,598)
HCPF	7.51 (4.78-9.70)	7.12 (4.59-9.46)	7.72 (4.91-9.82)
Q1	2.98 (2.17-3.65)	3.07 (2.28-3.68)	2.93 (2.12-3.62)
Q2	5.31 (4.78-5.89)	5.28 (4.76-5.85)	5.33 (4.79-5.91)
Q3	7.51 (7.00-7.98)	7.50 (6.98-7.96)	7.52 (7.01-7.99)
Q4	9.28 (8.87-9.70)	9.29 (8.88-9.70)	9.27 (8.86-9.70)
Q5	11.20 (10.60-12.10)	11.20 (10.60-12.10)	11.20 (10.60-12.10)
FD	12.72 (9.20-16.06)	11.79 (8.25-14.99)	13.26 (9.81-16.54)
Q1	5.87 (4.06-7.19)	5.75 (3.84-7.14)	5.98 (4.26-7.23)
Q2	10.10 (9.20-10.70)	10.00 (9.17-10.70)	10.10 (9.22-10.80)
Q3	12.70 (12.10-13.30)	12.70 (12.10-13.30)	12.70 (12.10-13.40)
Q4	15.30 (14.60-16.10)	15.20 (14.50-16.00)	15.30 (14.60-16.10)
Q5	18.80 (17.70-20.20)	18.70 (17.60-20.10)	18.90 (17.70-20.30)
HPGT	4.95 (3.31-6.66)	5.64 (4.05-7.31)	4.56 (2.98-6.24)
Q1	1.96 (1.23-2.50)	2.14 (1.50-2.60)	1.91 (1.16-2.46)
Q2	3.66 (3.31-4.00)	3.71 (3.34-4.03)	3.65 (3.30-3.98)
Q3	4.95 (4.63-5.26)	4.97 (4.65-5.28)	4.93 (4.63-5.26)
Q4	6.28 (5.92-6.66)	6.31 (5.94-6.68)	6.26 (5.91-6.64)
Q5	8.12 (7.54-8.94)	8.28 (7.62-9.15)	7.99 (7.48-8.71)
RHCB	5.71 (3.68-7.43)	6.33 (4.11-8.35)	5.41 (3.54-7.03)
Q1	2.81 (2.77-3.10)	2.77 (2.77-3.10)	2.81 (2.77-3.10)
Q2	4.11 (3.68-4.62)	4.14 (3.68-4.64)	4.11 (3.68-4.61)
Q3	5.71 (5.38-6.05)	5.71 (5.38-6.09)	5.71 (5.37-6.04)
Q4	7.00 (6.65-7.43)	6.98 (6.60-7.47)	7.00 (6.67-7.43)
Q5	9.58 (8.74-10.8)	9.78 (8.85-11.20)	9.40 (8.63-10.50)
VEG	7.94 (5.72-10.23)	7.66 (5.43-9.96)	8.08 (5.88-10.38)
Q1	3.87 (2.90-4.60)	3.82 (2.82-4.60)	3.90 (2.95-4.61)
Q2	6.22 (5.72-6.68)	6.20 (5.70-6.67)	6.22 (5.73-6.68)
Q3	7.94 (7.52-8.36)	7.94 (7.52-8.35)	7.94 (7.53-8.37)
Q4	9.72 (9.25-10.20)	9.70 (9.24-10.20)	9.72 (9.25-10.20)
Q5	12.40 (11.50-13.70)	12.40 (11.50-13.70)	12.40 (11.50-13.70)

HCPF (High Calorie and Processed Food) includes breads, chocolate/sweets, eggs, blue fish, white fish, soybean based food, milk, and pizza/hamburger

FD (Fruits and Dairy) includes yellow/orange fruit, white fruit, purple fruit, red/pink fruit, red vegetable, dairy

HPGT (High Protein and Green Tea) includes white meat, sushi, meat soup, unprocessed red meat, noodles, green tea drink

RHCB (Rice and High Calorie Beverages) includes soda drinks, other drinks, rice cake, white rice, mixed rice

VEG (Vegetables) includes mushroom, other vegetables, kimchi, carrot, brown vegetable, cruciferous vegetable, pumpkin, radish, allium vegetable, green vegetable, potatoes/starch

Supplementary Table S4. Hazard ratios for individual food group networks scores and risk of stroke or myocardial infarction

Network Scores: HR (95% CI)	Total Population (n=84,729)				Males (n=30,131)				Females (n=54,598)				p for interaction
	Cases	Model 1	Model 2	Model 3	Cases	Model 1	Model 2	Model 3	Cases	Model 1	Model 2	Model 3	
Cereal	3,097	0.99 (0.94-1.04)	1.02 (0.97-1.07)	1.02 (0.97–1.08)	1,482	0.98 (0.92-1.06)	0.99 (0.93-1.07)	0.99 (0.92–1.07)	1,615	1.00 (0.93-1.08)	1.05 (0.98-1.13)	1.07 (0.99–1.15)	0.638
Q1	295	1.00 (ref)	1.00 (ref)	1.00 (ref)	151	1.00 (ref)	1.00 (ref)	1.00 (ref)	144	1.00 (ref)	1.00 (ref)	1.00 (ref)	
Q2	307	1.04 (0.89-1.22)	1.04 (0.89-1.22)	1.04 (0.89–1.22)	160	1.08 (0.87-1.35)	1.08 (0.87-1.35)	1.09 (0.87–1.36)	147	1.01 (0.80-1.27)	1.00 (0.80-1.26)	1.00 (0.79–1.25)	
Q3	725	1.49 (1.30-1.71)	1.20 (1.03-1.40)	1.21 (1.04–1.42)	315	1.28 (1.05-1.55)	1.19 (0.96-1.48)	1.21 (0.97–1.51)	410	1.70 (1.40-2.06)	1.19 (0.96-1.49)	1.20 (0.97–1.50)	
Q4	1163	1.15 (1.00-1.32)	0.99 (0.85-1.15)	0.98 (0.85–1.15)	563	0.99 (0.82-1.21)	0.99 (0.80-1.22)	0.98 (0.79–1.21)	600	1.28 (1.06-1.56)	0.96 (0.77-1.19)	0.95 (0.77–1.18)	
Q5	607	1.25 (1.09-1.45)	1.14 (0.99-1.33)	1.15 (0.99–1.33)	293	1.12 (0.91-1.36)	1.11 (0.90-1.37)	1.10 (0.89–1.36)	314	1.40 (1.14-1.71)	1.17 (0.95-1.45)	1.19 (0.97–1.48)	
Drinks: Coffee	3,097	1.10 (1.06-1.14)	1.06 (1.03-1.10)	1.06 (1.02–1.10)	1,482	1.11 (1.05-1.16)	1.09 (1.04-1.15)	1.08 (1.02–1.13)	1,615	1.10 (1.05-1.15)	1.04 (0.99-1.09)	1.05 (1.00–1.10)	0.646
Q1	687	1.00 (ref)	1.00 (ref)	1.00 (ref)	228	1.00 (ref)	1.00 (ref)	1.00 (ref)	459	1.00 (ref)	1.00 (ref)	1.00 (ref)	
Q2	523	0.99 (0.88-1.11)	0.99 (0.89-1.11)	1.00 (0.89–1.12)	223	1.04 (0.86-1.25)	1.01 (0.84-1.21)	1.00 (0.83–1.21)	300	0.99 (0.86-1.15)	1.03 (0.89-1.19)	1.04 (0.90–1.20)	
Q3	630	0.99 (0.88-1.10)	0.98 (0.88-1.10)	0.98 (0.88–1.10)	309	1.07 (0.90-1.27)	1.06 (0.89-1.26)	1.03 (0.87–1.23)	321	0.94 (0.82-1.09)	0.95 (0.82-1.10)	0.95 (0.83–1.10)	
Q4	461	1.07 (0.95-1.21)	1.08 (0.95-1.21)	1.07 (0.94–1.20)	242	1.22 (1.02-1.47)	1.21 (1.00-1.45)	1.17 (0.97–1.40)	219	0.98 (0.83-1.15)	1.00 (0.85-1.18)	1.00 (0.84–1.18)	
Q5	796	1.32 (1.19-1.46)	1.22 (1.09-1.35)	1.20 (1.07–1.33)	480	1.34 (1.15-1.57)	1.30 (1.11-1.53)	1.24 (1.05–1.46)	316	1.36 (1.18-1.57)	1.17 (1.00-1.36)	1.18 (1.02–1.38)	
Legume	3,097	0.97 (0.93-1.02)	0.98 (0.94-1.02)	0.98 (0.94–1.03)	1,482	0.97 (0.91-1.04)	0.98 (0.91-1.04)	0.98 (0.91–1.04)	1,615	0.98 (0.92-1.04)	0.98 (0.92-1.04)	0.99 (0.93–1.05)	0.689
Q1	310	1.00 (ref)	1.00 (ref)	1.00 (ref)	168	1.00 (ref)	1.00 (ref)	1.00 (ref)	142	1.00 (ref)	1.00 (ref)	1.00 (ref)	
Q2	654	1.38 (1.20-1.58)	1.12 (0.97-1.31)	1.13 (0.97–1.31)	275	1.09 (0.90-1.33)	1.00 (0.81-1.24)	1.01 (0.82–1.25)	379	1.69 (1.39-2.06)	1.26 (1.01-1.56)	1.26 (1.02–1.56)	
Q3	825	1.20 (1.05-1.38)	1.06 (0.92-1.22)	1.06 (0.92–1.22)	392	0.98 (0.81-1.18)	0.96 (0.79-1.17)	0.95 (0.78–1.16)	433	1.45 (1.19-1.77)	1.17 (0.95-1.44)	1.17 (0.95–1.45)	
Q4	553	1.08 (0.94-1.25)	0.98 (0.84-1.13)	0.98 (0.84–1.13)	282	0.88 (0.73-1.07)	0.86 (0.70-1.05)	0.86 (0.70–1.05)	271	1.30 (1.06-1.60)	1.11 (0.89-1.37)	1.11 (0.90–1.38)	
Q5	755	1.14 (0.99-1.31)	1.02 (0.88-1.18)	1.03 (0.89–1.19)	365	0.96 (0.80-1.16)	0.94 (0.77-1.14)	0.94 (0.77–1.15)	390	1.33 (1.09-1.63)	1.11 (0.90-1.37)	1.13 (0.92–1.40)	
Meat: Red/Processed	3,097	0.98 (0.91-1.05)	1.01 (0.94-1.08)	1.01 (0.94–1.09)	1,482	0.98 (0.89-1.08)	0.99 (0.90-1.09)	0.99 (0.90–1.09)	1,615	0.98 (0.88-1.09)	1.03 (0.92-1.15)	1.04 (0.93–1.15)	0.643
Q1	306	1.00 (ref)	1.00 (ref)	1.00 (ref)	149	1.00 (ref)	1.00 (ref)	1.00 (ref)	157	1.00 (ref)	1.00 (ref)	1.00 (ref)	
Q2	350	1.14 (0.97-1.32)	1.13 (0.97-1.32)	1.13 (0.97–1.32)	185	1.24 (1.00-1.54)	1.24 (1.00-1.54)	1.24 (1.00–1.54)	165	1.04 (0.84-1.29)	1.03 (0.83-1.28)	1.02 (0.82–1.27)	
Q3	530	1.32 (1.15-1.52)	1.10 (0.94-1.28)	1.10 (0.94–1.28)	232	1.18 (0.96-1.45)	1.08 (0.86-1.34)	1.08 (0.87–1.34)	298	1.45 (1.19-1.76)	1.10 (0.89-1.36)	1.10 (0.89–1.35)	
Q4	1407	1.32 (1.16-1.51)	1.01 (0.86-1.18)	0.99 (0.85–1.16)	622	1.06 (0.88-1.28)	0.95 (0.77-1.19)	0.94 (0.75–1.17)	785	1.56 (1.30-1.87)	1.01 (0.81-1.26)	0.99 (0.80–1.24)	
Q5	504	1.26 (1.09-1.46)	1.08 (0.92-1.26)	1.08 (0.92–1.26)	294	1.13 (0.92-1.39)	1.06 (0.85-1.31)	1.05 (0.84–1.30)	210	1.32 (1.07-1.63)	1.06 (0.84-1.33)	1.06 (0.84–1.33)	
Nuts	3,097	0.99 (0.93-1.05)	1.04 (0.99-1.11)	1.06 (1.00–1.13)	1,482	1.05 (0.97-1.13)	1.05 (0.97-1.14)	1.06 (0.98–1.15)	1,615	0.95 (0.87-1.03)	1.06 (0.97-1.15)	1.09 (1.00–1.19)	0.185
Q1	345	1.00 (ref)	1.00 (ref)	1.00 (ref)	185	1.00 (ref)	1.00 (ref)	1.00 (ref)	160	1.00 (ref)	1.00 (ref)	1.00 (ref)	
Q2	702	1.33 (1.17-1.51)	1.07 (0.92-1.23)	1.07 (0.92–1.24)	285	1.04 (0.86-1.25)	0.94 (0.76-1.15)	0.94 (0.77–1.16)	417	1.64 (1.36-1.97)	1.19 (0.96-1.46)	1.19 (0.97–1.47)	
Q3	1042	1.04 (0.91-1.18)	0.89 (0.78-1.03)	0.88 (0.77–1.02)	462	0.82 (0.69-0.98)	0.79 (0.65-0.96)	0.78 (0.64–0.95)	580	1.27 (1.06-1.53)	0.99 (0.81-1.21)	0.98 (0.80–1.20)	

Network Scores: HR (95% CI)	Total Population (n=84,729)				Males (n=30,131)				Females (n=54,598)				p for interaction
	Cases	Model 1	Model 2	Model 3	Cases	Model 1	Model 2	Model 3	Cases	Model 1	Model 2	Model 3	
Q4	527	1.10 (0.96-1.26)	1.01 (0.87-1.16)	1.02 (0.88–1.18)	291	0.96 (0.79-1.15)	0.92 (0.76-1.12)	0.93 (0.76–1.13)	236	1.25 (1.02-1.53)	1.10 (0.89-1.35)	1.12 (0.91–1.38)	
Q5	481	1.07 (0.93-1.23)	1.00 (0.86-1.16)	1.02 (0.88–1.18)	259	0.96 (0.79-1.16)	0.92 (0.75-1.12)	0.92 (0.75–1.13)	222	1.19 (0.97-1.46)	1.11 (0.89-1.37)	1.15 (0.92–1.42)	
Seafood: Crustacean	3,097	0.92 (0.87-0.97)	0.95 (0.90-1.00)	0.95 (0.90–1.00)	1,482	0.92 (0.85-0.99)	0.92 (0.85-0.99)	0.91 (0.84–0.98)	1,615	0.92 (0.85-0.99)	0.97 (0.90-1.05)	0.99 (0.91–1.07)	0.773
Q1	327	1.00 (ref)	1.00 (ref)	1.00 (ref)	162	1.00 (ref)	1.00 (ref)	1.00 (ref)	165	1.00 (ref)	1.00 (ref)	1.00 (ref)	
Q2	669	1.35 (1.18-1.54)	1.09 (0.94-1.27)	1.10 (0.95–1.27)	283	1.16 (0.95-1.41)	1.07 (0.87-1.32)	1.08 (0.87–1.33)	386	1.51 (1.25-1.81)	1.10 (0.89-1.35)	1.10 (0.90–1.35)	
Q3	1058	1.10 (0.96-1.25)	0.94 (0.81-1.08)	0.93 (0.81–1.07)	487	0.96 (0.80-1.16)	0.95 (0.78-1.16)	0.94 (0.77–1.15)	571	1.20 (1.00-1.44)	0.91 (0.74-1.11)	0.90 (0.73–1.10)	
Q4	493	1.03 (0.90-1.19)	0.95 (0.82-1.09)	0.95 (0.82–1.10)	251	0.92 (0.76-1.13)	0.90 (0.73-1.11)	0.89 (0.73–1.10)	242	1.12 (0.92-1.37)	0.98 (0.80-1.20)	0.99 (0.80–1.21)	
Q5	550	1.00 (0.87-1.15)	0.90 (0.78-1.05)	0.91 (0.78–1.05)	299	0.89 (0.73-1.08)	0.86 (0.70-1.06)	0.85 (0.69–1.04)	251	1.08 (0.89-1.32)	0.93 (0.75-1.14)	0.94 (0.76–1.16)	
Seafood: Mollusks	3,097	0.87 (0.81-0.93)	0.91 (0.86-0.98)	0.92 (0.86–0.99)	1,482	0.92 (0.84-1.01)	0.92 (0.84-1.01)	0.91 (0.83–1.00)	1,615	0.82 (0.75-0.90)	0.93 (0.84-1.02)	0.95 (0.86–1.04)	0.073
Q1	873	1.00 (ref)	1.00 (ref)	1.00 (ref)	316	1.00 (ref)	1.00 (ref)	1.00 (ref)	557	1.00 (ref)	1.00 (ref)	1.00 (ref)	
Q2	625	0.88 (0.80-0.98)	0.93 (0.83-1.03)	0.94 (0.85–1.04)	294	0.99 (0.85-1.17)	1.01 (0.86-1.18)	1.02 (0.87–1.20)	331	0.83 (0.72-0.95)	0.89 (0.78-1.02)	0.90 (0.79–1.04)	
Q3	554	0.84 (0.76-0.94)	0.91 (0.82-1.02)	0.93 (0.83–1.04)	270	0.90 (0.76-1.06)	0.92 (0.78-1.09)	0.93 (0.79–1.10)	284	0.82 (0.71-0.95)	0.94 (0.81-1.09)	0.96 (0.82–1.11)	
Q4	505	0.82 (0.73-0.92)	0.89 (0.79-1.00)	0.90 (0.80–1.01)	296	1.00 (0.85-1.18)	1.00 (0.85-1.18)	1.01 (0.85–1.19)	209	0.69 (0.59-0.81)	0.81 (0.68-0.95)	0.83 (0.70–0.98)	
Q5	540	0.79 (0.71-0.88)	0.86 (0.77-0.97)	0.88 (0.78–0.99)	306	0.88 (0.75-1.03)	0.87 (0.74-1.03)	0.87 (0.74–1.04)	234	0.74 (0.63-0.87)	0.90 (0.76-1.06)	0.93 (0.78–1.10)	
Seafood: Processed	3,097	0.98 (0.92-1.04)	0.99 (0.93-1.05)	0.99 (0.93–1.06)	1,482	0.93 (0.85-1.01)	0.92 (0.84-1.01)	0.91 (0.83–1.00)	1,615	1.02 (0.94-1.11)	1.05 (0.97-1.14)	1.06 (0.97–1.15)	0.202
Q1	356	1.00 (ref)	1.00 (ref)	1.00 (ref)	168	1.00 (ref)	1.00 (ref)	1.00 (ref)	188	1.00 (ref)	1.00 (ref)	1.00 (ref)	
Q2	1067	1.25 (1.10-1.41)	0.99 (0.86-1.13)	0.97 (0.85–1.12)	451	1.04 (0.87-1.25)	0.98 (0.80-1.19)	0.96 (0.78–1.18)	616	1.41 (1.19-1.66)	0.97 (0.80-1.18)	0.95 (0.79–1.16)	
Q3	543	1.08 (0.94-1.23)	0.94 (0.81-1.08)	0.94 (0.81–1.08)	286	1.04 (0.86-1.26)	1.01 (0.82-1.23)	1.00 (0.82–1.23)	257	1.07 (0.88-1.29)	0.86 (0.70-1.05)	0.86 (0.70–1.05)	
Q4	499	1.10 (0.96-1.27)	0.96 (0.83-1.11)	0.96 (0.83–1.11)	260	1.00 (0.82-1.22)	0.96 (0.78-1.18)	0.94 (0.77–1.16)	239	1.17 (0.97-1.42)	0.94 (0.77-1.16)	0.95 (0.77–1.16)	
Q5	632	1.15 (1.00-1.31)	1.00 (0.87-1.15)	0.99 (0.86–1.15)	317	0.95 (0.78-1.15)	0.91 (0.74-1.11)	0.88 (0.72–1.08)	315	1.33 (1.11-1.60)	1.07 (0.88-1.30)	1.08 (0.89–1.32)	
Seaweed	3,097	0.97 (0.88-1.06)	1.01 (0.92-1.11)	1.04 (0.94–1.14)	1,482	1.06 (0.93-1.22)	1.04 (0.91-1.20)	1.04 (0.90–1.21)	1,615	0.91 (0.80-1.02)	1.00 (0.89-1.13)	1.05 (0.92–1.19)	0.108
Q1	741	1.00 (ref)	1.00 (ref)	1.00 (ref)	376	1.00 (ref)	1.00 (ref)	1.00 (ref)	365	1.00 (ref)	1.00 (ref)	1.00 (ref)	
Q2	627	1.00 (0.90-1.12)	1.03 (0.93-1.15)	1.05 (0.94–1.17)	314	1.01 (0.87-1.17)	1.01 (0.87-1.18)	1.02 (0.88–1.19)	313	0.99 (0.85-1.15)	1.05 (0.90-1.22)	1.07 (0.92–1.24)	
Q3	504	0.94 (0.84-1.06)	0.99 (0.88-1.11)	1.01 (0.90–1.13)	229	0.96 (0.81-1.13)	0.95 (0.80-1.12)	0.96 (0.81–1.13)	275	0.93 (0.79-1.09)	1.02 (0.87-1.19)	1.05 (0.89–1.23)	
Q4	647	0.92 (0.83-1.03)	0.97 (0.87-1.08)	0.99 (0.89–1.11)	318	0.98 (0.84-1.14)	0.98 (0.84-1.14)	0.99 (0.84–1.16)	329	0.87 (0.75-1.01)	0.95 (0.81-1.10)	0.99 (0.84–1.15)	
Q5	578	0.99 (0.89-1.11)	1.05 (0.94-1.17)	1.08 (0.96–1.22)	245	1.10 (0.93-1.29)	1.07 (0.91-1.26)	1.07 (0.90–1.27)	333	0.92 (0.79-1.07)	1.05 (0.90-1.22)	1.11 (0.95–1.30)	

HR: Hazard ratio; CI: confidence interval
Model 1: adjusted for age and sex (for total respondents only)
Model 2: adjusted for education, city, marriage status, income group, job group, previous history of hypertension, hyperlipidemia, or diabetes, BMI, and waist circumference, added to Model 1
Model 3: adjusted for drinking status, smoking status, exercise, and total energy intake, added to Model 2
p for interaction was computed using Model 3 with interaction term of sex*food group

Supplementary Table S5. Hazard ratios for individual food group networks scores and risk of stroke

Network Scores: HR (95% CI)	Total Population (n=84,729)				Males (n=30,131)				Females (n=54,598)				p for interaction
	Cases	Model 1	Model 2	Model 3	Cases	Model 1	Model 2	Model 3	Cases	Model 1	Model 2	Model 3	
Cereal	1,040	0.95 (0.87-1.04)	0.99 (0.90-1.08)	0.99 (0.90–1.09)	512	0.93 (0.82-1.06)	0.95 (0.83-1.08)	0.95 (0.83–1.08)	528	0.97 (0.85-1.11)	1.03 (0.90-1.18)	1.05 (0.91–1.20)	0.483
Q1	80	1.00 (ref)	1.00 (ref)	1.00 (ref)	36	1.00 (ref)	1.00 (ref)	1.00 (ref)	44	1.00 (ref)	1.00 (ref)	1.00 (ref)	
Q2	74	0.93 (0.68-1.27)	0.91 (0.67-1.26)	0.92 (0.67–1.26)	39	1.10 (0.70-1.73)	1.09 (0.69-1.71)	1.10 (0.70–1.73)	35	0.78 (0.50-1.22)	0.77 (0.50-1.21)	0.77 (0.49–1.20)	
Q3	226	1.72 (1.33-2.22)	1.20 (0.90-1.60)	1.20 (0.90–1.60)	109	1.84 (1.26-2.68)	1.49 (0.98-2.26)	1.50 (0.98–2.28)	117	1.61 (1.13-2.29)	0.96 (0.64-1.43)	0.96 (0.64–1.43)	
Q4	469	1.44 (1.11-1.86)	1.07 (0.81-1.41)	1.06 (0.80–1.40)	238	1.64 (1.13-2.39)	1.40 (0.93-2.09)	1.37 (0.92–2.06)	231	1.25 (0.88-1.78)	0.80 (0.54-1.19)	0.80 (0.54–1.18)	
Q5	191	1.35 (1.03-1.76)	1.10 (0.83-1.46)	1.10 (0.83–1.46)	90	1.40 (0.95-2.07)	1.24 (0.82-1.87)	1.23 (0.81–1.85)	101	1.30 (0.91-1.87)	0.97 (0.66-1.43)	0.98 (0.67–1.45)	
Drinks: Coffee	1,040	1.14 (1.07-1.21)	1.09 (1.03-1.16)	1.09 (1.03–1.16)	512	1.15 (1.06-1.25)	1.12 (1.03-1.22)	1.11 (1.02–1.21)	528	1.13 (1.04-1.23)	1.07 (0.98-1.16)	1.08 (0.99–1.18)	0.571
Q1	230	1.00 (ref)	1.00 (ref)	1.00 (ref)	80	1.00 (ref)	1.00 (ref)	1.00 (ref)	150	1.00 (ref)	1.00 (ref)	1.00 (ref)	
Q2	156	0.91 (0.74-1.11)	0.94 (0.77-1.15)	0.95 (0.77–1.16)	72	0.97 (0.71-1.34)	0.99 (0.72-1.36)	0.99 (0.72–1.36)	84	0.88 (0.67-1.15)	0.93 (0.71-1.22)	0.94 (0.72–1.24)	
Q3	230	1.07 (0.89-1.29)	1.08 (0.89-1.29)	1.08 (0.89–1.29)	112	1.12 (0.84-1.50)	1.12 (0.84-1.49)	1.10 (0.82–1.47)	118	1.04 (0.81-1.32)	1.06 (0.83-1.35)	1.07 (0.84–1.37)	
Q4	143	1.10 (0.89-1.37)	1.15 (0.92-1.42)	1.14 (0.92–1.41)	72	1.12 (0.81-1.55)	1.15 (0.83-1.59)	1.13 (0.81–1.56)	71	1.11 (0.83-1.48)	1.15 (0.86-1.54)	1.17 (0.87–1.56)	
Q5	281	1.46 (1.22-1.75)	1.34 (1.12-1.62)	1.33 (1.11–1.60)	176	1.48 (1.13-1.93)	1.41 (1.08-1.85)	1.37 (1.04–1.80)	105	1.49 (1.16-1.92)	1.28 (0.98-1.67)	1.32 (1.01–1.72)	
Legume	1,040	0.96 (0.89-1.03)	0.95 (0.89-1.03)	0.96 (0.89–1.03)	512	0.95 (0.85-1.06)	0.94 (0.84-1.05)	0.94 (0.84–1.05)	528	0.96 (0.87-1.06)	0.96 (0.87-1.06)	0.97 (0.88–1.07)	0.482
Q1	76	1.00 (ref)	1.00 (ref)	1.00 (ref)	37	1.00 (ref)	1.00 (ref)	1.00 (ref)	39	1.00 (ref)	1.00 (ref)	1.00 (ref)	
Q2	203	1.73 (1.33-2.26)	1.24 (0.93-1.65)	1.23 (0.92–1.65)	94	1.65 (1.13-2.42)	1.29 (0.86-1.96)	1.29 (0.85–1.95)	109	1.81 (1.25-2.62)	1.18 (0.79-1.77)	1.18 (0.79–1.77)	
Q3	326	1.66 (1.28-2.15)	1.29 (0.99-1.70)	1.29 (0.98–1.69)	153	1.57 (1.08-2.27)	1.33 (0.90-1.96)	1.31 (0.89–1.94)	173	1.73 (1.20-2.48)	1.25 (0.85-1.84)	1.26 (0.86–1.84)	
Q4	176	1.25 (0.95-1.65)	1.01 (0.76-1.34)	1.01 (0.76–1.34)	100	1.33 (0.91-1.95)	1.14 (0.77-1.70)	1.14 (0.76–1.69)	76	1.15 (0.77-1.70)	0.88 (0.58-1.32)	0.88 (0.59–1.33)	
Q5	259	1.37 (1.05-1.78)	1.08 (0.82-1.43)	1.09 (0.83–1.44)	128	1.38 (0.95-2.01)	1.16 (0.78-1.72)	1.16 (0.78–1.72)	131	1.34 (0.92-1.94)	1.00 (0.68-1.48)	1.01 (0.68–1.50)	
Meat: Red/Processed	1,040	0.94 (0.83-1.06)	0.97 (0.86-1.10)	0.97 (0.86–1.10)	512	0.89 (0.75-1.06)	0.92 (0.77-1.09)	0.91 (0.77–1.08)	528	0.99 (0.83-1.19)	1.04 (0.87-1.25)	1.05 (0.88–1.26)	0.343
Q1	74	1.00 (ref)	1.00 (ref)	1.00 (ref)	33	1.00 (ref)	1.00 (ref)	1.00 (ref)	41	1.00 (ref)	1.00 (ref)	1.00 (ref)	
Q2	87	1.17 (0.86-1.59)	1.16 (0.85-1.58)	1.16 (0.85–1.58)	48	1.45 (0.93-2.26)	1.45 (0.93-2.25)	1.46 (0.94–2.28)	39	0.94 (0.61-1.46)	0.92 (0.60-1.43)	0.92 (0.59–1.42)	
Q3	164	1.69 (1.29-2.23)	1.30 (0.97-1.75)	1.30 (0.97–1.74)	84	1.92 (1.28-2.87)	1.61 (1.05-2.47)	1.61 (1.05–2.47)	80	1.51 (1.03-2.20)	1.05 (0.70-1.58)	1.05 (0.70–1.57)	
Q4	541	1.82 (1.41-2.36)	1.22 (0.91-1.63)	1.19 (0.89–1.59)	253	1.79 (1.22-2.61)	1.37 (0.89-2.09)	1.33 (0.87–2.03)	288	1.84 (1.30-2.61)	1.06 (0.71-1.58)	1.04 (0.69–1.56)	
Q5	174	1.63 (1.23-2.16)	1.27 (0.95-1.72)	1.26 (0.93–1.70)	94	1.59 (1.06-2.39)	1.36 (0.88-2.08)	1.33 (0.86–2.04)	80	1.66 (1.13-2.45)	1.19 (0.79-1.81)	1.19 (0.78–1.81)	
Nuts	1,040	0.92 (0.83-1.02)	1.00 (0.90-1.11)	1.02 (0.92–1.13)	512	0.98 (0.85-1.12)	1.01 (0.88-1.17)	1.02 (0.88–1.18)	528	0.87 (0.75-1.02)	1.01 (0.86-1.18)	1.04 (0.89–1.22)	0.488
Q1	89	1.00 (ref)	1.00 (ref)	1.00 (ref)	49	1.00 (ref)	1.00 (ref)	1.00 (ref)	40	1.00 (ref)	1.00 (ref)	1.00 (ref)	
Q2	215	1.56 (1.22-2.01)	1.08 (0.82-1.43)	1.08 (0.82–1.42)	93	1.25 (0.88-1.77)	0.92 (0.63-1.36)	0.93 (0.63–1.36)	122	1.96 (1.36-2.81)	1.26 (0.85-1.89)	1.27 (0.85–1.89)	
Q3	402	1.27 (0.99-1.62)	0.96 (0.74-1.25)	0.95 (0.73–1.23)	183	1.07 (0.77-1.50)	0.86 (0.60-1.23)	0.84 (0.59–1.21)	219	1.49 (1.04-2.14)	1.07 (0.72-1.57)	1.05 (0.72–1.55)	

Network Scores: HR (95% CI)	**Total Population (n=84,729)**				**Males (n=30,131)**				**Females (n=54,598)**				**p for interaction**
	Cases	Model 1	Model 2	Model 3	Cases	Model 1	Model 2	Model 3	Cases	Model 1	Model 2	Model 3	
Q4	176	1.29 (1.00-1.67)	1.07 (0.82-1.40)	1.08 (0.83–1.42)	96	1.11 (0.79-1.58)	0.95 (0.66-1.37)	0.95 (0.66–1.37)	80	1.50 (1.02-2.20)	1.23 (0.82-1.83)	1.25 (0.84–1.87)	
Q5	158	1.22 (0.94-1.59)	1.04 (0.79-1.37)	1.06 (0.80–1.40)	91	1.17 (0.82-1.66)	0.98 (0.67-1.42)	0.98 (0.67–1.43)	67	1.27 (0.86-1.89)	1.13 (0.75-1.71)	1.18 (0.78–1.78)	
Seafood: Crustacean	1,040	0.89 (0.82-0.98)	0.93 (0.85-1.03)	0.94 (0.85–1.03)	512	0.90 (0.79-1.02)	0.92 (0.81-1.04)	0.91 (0.80–1.04)	528	0.89 (0.78-1.02)	0.95 (0.83-1.09)	0.97 (0.84–1.11)	0.990
Q1	83	1.00 (ref)	1.00 (ref)	1.00 (ref)	39	1.00 (ref)	1.00 (ref)	1.00 (ref)	44	1.00 (ref)	1.00 (ref)	1.00 (ref)	
Q2	200	1.58 (1.22-2.04)	1.13 (0.85-1.50)	1.13 (0.85–1.50)	90	1.51 (1.03-2.20)	1.19 (0.79-1.79)	1.18 (0.78–1.78)	110	1.64 (1.15-2.33)	1.07 (0.72-1.58)	1.07 (0.72–1.58)	
Q3	420	1.45 (1.13-1.86)	1.11 (0.85-1.45)	1.09 (0.84–1.43)	202	1.51 (1.05-2.16)	1.28 (0.87-1.88)	1.25 (0.85–1.84)	218	1.37 (0.97-1.94)	0.95 (0.65-1.38)	0.94 (0.65–1.37)	
Q4	155	1.17 (0.89-1.54)	0.99 (0.75-1.31)	0.99 (0.75–1.31)	84	1.24 (0.84-1.82)	1.11 (0.75-1.65)	1.10 (0.74–1.63)	71	1.08 (0.74-1.59)	0.88 (0.59-1.30)	0.88 (0.59–1.31)	
Q5	182	1.17 (0.90-1.53)	0.97 (0.73-1.27)	0.96 (0.73–1.27)	97	1.15 (0.78-1.68)	1.00 (0.67-1.48)	0.96 (0.65–1.44)	85	1.19 (0.82-1.73)	0.94 (0.64-1.38)	0.96 (0.65–1.41)	
Seafood: Mollusks	1,040	0.89 (0.79-0.99)	0.95 (0.85-1.06)	0.96 (0.86–1.08)	512	0.94 (0.81-1.09)	0.96 (0.83-1.12)	0.96 (0.82–1.13)	528	0.84 (0.71-0.99)	0.96 (0.81-1.13)	0.98 (0.83–1.17)	0.682
Q1	282	1.00 (ref)	1.00 (ref)	1.00 (ref)	111	1.00 (ref)	1.00 (ref)	1.00 (ref)	171	1.00 (ref)	1.00 (ref)	1.00 (ref)	
Q2	206	0.90 (0.75-1.08)	0.97 (0.80-1.16)	0.98 (0.82–1.17)	98	0.96 (0.73-1.26)	1.01 (0.77-1.33)	1.02 (0.78–1.35)	108	0.86 (0.68-1.10)	0.94 (0.73-1.20)	0.95 (0.75–1.22)	
Q3	203	0.94 (0.78-1.13)	1.06 (0.88-1.28)	1.08 (0.90–1.31)	97	0.94 (0.72-1.25)	1.03 (0.77-1.36)	1.05 (0.79–1.39)	106	0.96 (0.75-1.23)	1.11 (0.86-1.43)	1.14 (0.88–1.47)	
Q4	153	0.77 (0.63-0.94)	0.87 (0.71-1.07)	0.88 (0.72–1.09)	96	0.95 (0.72-1.26)	1.03 (0.77-1.36)	1.03 (0.78–1.38)	57	0.59 (0.44-0.80)	0.71 (0.52-0.97)	0.73 (0.54–1.01)	
Q5	196	0.86 (0.71-1.04)	0.98 (0.80-1.20)	1.01 (0.82–1.24)	110	0.92 (0.70-1.20)	0.98 (0.74-1.30)	0.98 (0.73–1.32)	86	0.83 (0.64-1.09)	1.03 (0.78-1.37)	1.08 (0.81–1.45)	
Seafood: Processed	1,040	1.01 (0.91-1.12)	1.02 (0.92-1.13)	1.02 (0.92–1.14)	512	0.92 (0.79-1.07)	0.92 (0.79-1.07)	0.91 (0.77–1.06)	528	1.10 (0.95-1.26)	1.12 (0.97-1.28)	1.13 (0.99–1.31)	0.088
Q1	92	1.00 (ref)	1.00 (ref)	1.00 (ref)	45	1.00 (ref)	1.00 (ref)	1.00 (ref)	47	1.00 (ref)	1.00 (ref)	1.00 (ref)	
Q2	378	1.50 (1.18-1.89)	1.01 (0.78-1.32)	0.99 (0.76–1.29)	173	1.35 (0.96-1.89)	1.03 (0.71-1.50)	1.00 (0.69–1.45)	205	1.61 (1.16-2.24)	0.98 (0.68-1.42)	0.97 (0.67–1.41)	
Q3	176	1.21 (0.93-1.56)	0.94 (0.71-1.23)	0.93 (0.71–1.22)	99	1.27 (0.88-1.81)	1.06 (0.73-1.55)	1.04 (0.71–1.52)	77	1.10 (0.76-1.59)	0.80 (0.54-1.18)	0.80 (0.54–1.18)	
Q4	165	1.29 (1.00-1.67)	1.00 (0.77-1.32)	0.99 (0.76–1.31)	81	1.11 (0.77-1.61)	0.94 (0.64-1.38)	0.90 (0.61–1.33)	84	1.46 (1.01-2.09)	1.05 (0.72-1.55)	1.06 (0.72–1.56)	
Q5	229	1.42 (1.11-1.82)	1.08 (0.83-1.40)	1.07 (0.82–1.40)	114	1.20 (0.84-1.71)	0.98 (0.67-1.42)	0.93 (0.64–1.36)	115	1.64 (1.16-2.33)	1.17 (0.81-1.70)	1.20 (0.83–1.75)	
Seaweed	1,040	0.89 (0.76-1.05)	0.97 (0.83-1.14)	1.00 (0.84–1.18)	512	0.95 (0.75-1.20)	0.98 (0.77-1.25)	0.99 (0.77–1.28)	528	0.85 (0.69-1.05)	0.98 (0.79-1.21)	1.02 (0.81–1.28)	0.621
Q1	233	1.00 (ref)	1.00 (ref)	1.00 (ref)	123	1.00 (ref)	1.00 (ref)	1.00 (ref)	110	1.00 (ref)	1.00 (ref)	1.00 (ref)	
Q2	229	1.16 (0.97-1.40)	1.20 (1.00-1.45)	1.23 (1.02–1.47)	111	1.10 (0.85-1.43)	1.13 (0.87-1.46)	1.15 (0.88–1.49)	118	1.22 (0.94-1.58)	1.28 (0.99-1.67)	1.30 (1.00–1.70)	
Q3	168	1.03 (0.85-1.26)	1.10 (0.90-1.35)	1.13 (0.92–1.38)	83	1.09 (0.83-1.44)	1.13 (0.85-1.49)	1.14 (0.86–1.52)	85	0.97 (0.73-1.29)	1.09 (0.81-1.45)	1.12 (0.84–1.49)	
Q4	237	1.05 (0.87-1.25)	1.11 (0.92-1.34)	1.14 (0.95–1.38)	120	1.13 (0.87-1.45)	1.16 (0.90-1.51)	1.19 (0.91–1.55)	117	0.96 (0.74-1.25)	1.06 (0.81-1.38)	1.10 (0.84–1.44)	
Q5	173	0.95 (0.78-1.16)	1.06 (0.86-1.29)	1.10 (0.89–1.35)	75	1.04 (0.78-1.39)	1.09 (0.81-1.46)	1.11 (0.82–1.51)	98	0.89 (0.68-1.17)	1.05 (0.79-1.38)	1.10 (0.82–1.47)	

HR: Hazard ratio; CI: confidence interval
Model 1: adjusted for age and sex (for total respondents only)
Model 2: adjusted for education, city, marriage status, income group, job group, previous history of hypertension, hyperlipidemia, or diabetes, BMI, and waist circumference, added to Model 1
Model 3: adjusted for drinking status, smoking status, exercise, and total energy intake, added to Model 2
p for interaction was computed using Model 3 with interaction term of sex*food group

Supplementary Table S6. Hazard ratios for individual food group networks scores and risk of myocardial infarction

Network Scores: HR (95% CI)	Total Population (n=84,729)				Males (n=30,131)				Females (n=54,598)				p for interaction
	Cases	Model 1	Model 2	Model 3	Cases	Model 1	Model 2	Model 3	Cases	Model 1	Model 2	Model 3	
Cereal	2,001	1.01 (0.94-1.07)	1.03 (0.97-1.10)	1.03 (0.97–1.10)	947	1.01 (0.93-1.11)	1.02 (0.93-1.11)	1.01 (0.92–1.11)	1,054	1.01 (0.92-1.11)	1.06 (0.97-1.16)	1.07 (0.98–1.17)	0.985
Q1	210	1.00 (ref)	1.00 (ref)	1.00 (ref)	113	1.00 (ref)	1.00 (ref)	1.00 (ref)	97	1.00 (ref)	1.00 (ref)	1.00 (ref)	
Q2	229	1.09 (0.90-1.32)	1.10 (0.91-1.32)	1.10 (0.91–1.32)	120	1.09 (0.84-1.41)	1.09 (0.84-1.41)	1.09 (0.84–1.41)	109	1.11 (0.84-1.46)	1.11 (0.84-1.45)	1.10 (0.84–1.45)	
Q3	486	1.41 (1.20-1.66)	1.19 (0.99-1.43)	1.21 (1.00–1.45)	202	1.11 (0.88-1.40)	1.08 (0.83-1.40)	1.10 (0.85–1.43)	284	1.73 (1.37-2.19)	1.30 (1.00-1.69)	1.31 (1.00–1.71)	
Q4	674	1.03 (0.87-1.22)	0.93 (0.78-1.12)	0.93 (0.77–1.11)	314	0.77 (0.61-0.98)	0.82 (0.63-1.05)	0.81 (0.63–1.05)	360	1.30 (1.03-1.65)	1.01 (0.78-1.32)	1.01 (0.78–1.32)	
Q5	402	1.21 (1.02-1.44)	1.15 (0.96-1.37)	1.15 (0.96–1.38)	198	1.03 (0.81-1.30)	1.06 (0.83-1.36)	1.05 (0.82–1.34)	204	1.42 (1.11-1.82)	1.24 (0.96-1.61)	1.27 (0.98–1.64)	
Drinks: Coffee	2,001	1.08 (1.03-1.12)	1.04 (0.99-1.08)	1.03 (0.99–1.08)	947	1.09 (1.02-1.16)	1.08 (1.01-1.15)	1.06 (0.99–1.13)	1,054	1.07 (1.01-1.13)	1.01 (0.95-1.07)	1.02 (0.96–1.08)	0.607
Q1	445	1.00 (ref)	1.00 (ref)	1.00 (ref)	142	1.00 (ref)	1.00 (ref)	1.00 (ref)	303	1.00 (ref)	1.00 (ref)	1.00 (ref)	
Q2	362	1.04 (0.90-1.19)	1.02 (0.89-1.18)	1.03 (0.89–1.18)	149	1.10 (0.87-1.38)	1.04 (0.82-1.31)	1.03 (0.82–1.30)	213	1.05 (0.88-1.25)	1.08 (0.90-1.28)	1.08 (0.91–1.29)	
Q3	390	0.94 (0.82-1.08)	0.93 (0.81-1.07)	0.93 (0.81–1.07)	195	1.07 (0.86-1.33)	1.05 (0.85-1.31)	1.02 (0.82–1.27)	195	0.88 (0.73-1.05)	0.88 (0.73-1.05)	0.87 (0.73–1.05)	
Q4	309	1.05 (0.90-1.21)	1.04 (0.89-1.20)	1.02 (0.88–1.19)	167	1.30 (1.04-1.63)	1.25 (0.99-1.57)	1.20 (0.96–1.51)	142	0.91 (0.74-1.11)	0.92 (0.75-1.12)	0.91 (0.74–1.12)	
Q5	495	1.23 (1.08-1.40)	1.13 (0.99-1.29)	1.11 (0.97–1.27)	294	1.28 (1.05-1.57)	1.24 (1.01-1.52)	1.18 (0.96–1.45)	201	1.27 (1.06-1.52)	1.08 (0.90-1.31)	1.08 (0.90–1.31)	
Legume	2,001	0.99 (0.93-1.04)	0.99 (0.94-1.05)	1.00 (0.95–1.06)	947	0.98 (0.90-1.06)	0.99 (0.91-1.08)	0.99 (0.91–1.08)	1,054	0.99 (0.93-1.07)	0.99 (0.93-1.07)	1.00 (0.93–1.08)	0.819
Q1	229	1.00 (ref)	1.00 (ref)	1.00 (ref)	129	1.00 (ref)	1.00 (ref)	1.00 (ref)	100	1.00 (ref)	1.00 (ref)	1.00 (ref)	
Q2	435	1.25 (1.06-1.47)	1.07 (0.89-1.27)	1.07 (0.90–1.28)	179	0.94 (0.75-1.18)	0.92 (0.72-1.18)	0.93 (0.73–1.20)	256	1.61 (1.27-2.04)	1.24 (0.96-1.61)	1.25 (0.97–1.62)	
Q3	484	1.03 (0.88-1.22)	0.95 (0.80-1.12)	0.95 (0.80–1.13)	232	0.79 (0.64-0.99)	0.83 (0.66-1.05)	0.83 (0.65–1.05)	252	1.32 (1.04-1.68)	1.09 (0.85-1.40)	1.10 (0.85–1.41)	
Q4	368	1.03 (0.87-1.22)	0.97 (0.81-1.15)	0.97 (0.81–1.15)	178	0.75 (0.60-0.95)	0.77 (0.61-0.98)	0.77 (0.61–0.98)	190	1.38 (1.08-1.77)	1.21 (0.94-1.56)	1.22 (0.95–1.57)	
Q5	485	1.07 (0.91-1.26)	1.00 (0.84-1.18)	1.01 (0.85–1.20)	229	0.83 (0.67-1.04)	0.86 (0.68-1.09)	0.86 (0.68–1.09)	256	1.37 (1.08-1.73)	1.17 (0.91-1.50)	1.20 (0.93–1.54)	
Meat: Red/Processed	2,001	1.01 (0.92-1.10)	1.03 (0.94-1.12)	1.03 (0.94–1.13)	947	1.02 (0.91-1.14)	1.02 (0.91-1.14)	1.02 (0.91–1.15)	1,054	0.98 (0.85-1.12)	1.03 (0.90-1.18)	1.03 (0.90–1.19)	0.273
Q1	226	1.00 (ref)	1.00 (ref)	1.00 (ref)	112	1.00 (ref)	1.00 (ref)	1.00 (ref)	114	1.00 (ref)	1.00 (ref)	1.00 (ref)	
Q2	260	1.14 (0.96-1.36)	1.14 (0.95-1.36)	1.14 (0.96–1.37)	136	1.21 (0.95-1.56)	1.21 (0.94-1.56)	1.21 (0.95–1.56)	124	1.08 (0.83-1.39)	1.07 (0.83-1.38)	1.07 (0.83–1.37)	
Q3	350	1.18 (1.00-1.40)	1.00 (0.84-1.20)	1.00 (0.84–1.20)	144	0.98 (0.77-1.26)	0.92 (0.70-1.19)	0.92 (0.71–1.20)	206	1.37 (1.09-1.73)	1.06 (0.83-1.36)	1.06 (0.82–1.36)	
Q4	842	1.16 (0.99-1.35)	0.92 (0.76-1.10)	0.90 (0.75–1.09)	360	0.86 (0.69-1.08)	0.83 (0.64-1.08)	0.82 (0.63–1.07)	482	1.44 (1.16-1.78)	0.95 (0.73-1.23)	0.93 (0.71–1.21)	
Q5	323	1.15 (0.97-1.37)	1.00 (0.83-1.21)	1.00 (0.83–1.21)	195	1.02 (0.80-1.29)	0.97 (0.75-1.26)	0.97 (0.75–1.26)	128	1.20 (0.93-1.55)	0.98 (0.74-1.29)	0.97 (0.74–1.28)	
Nuts	2,001	1.02 (0.95-1.09)	1.06 (0.99-1.14)	1.07 (1.00–1.15)	947	1.08 (0.98-1.19)	1.07 (0.97-1.18)	1.07 (0.97–1.19)	1,054	0.98 (0.89-1.08)	1.08 (0.97-1.19)	1.10 (1.00–1.22)	0.251
Q1	250	1.00 (ref)	1.00 (ref)	1.00 (ref)	133	1.00 (ref)	1.00 (ref)	1.00 (ref)	117	1.00 (ref)	1.00 (ref)	1.00 (ref)	
Q2	472	1.24 (1.07-1.45)	1.05 (0.88-1.25)	1.06 (0.89–1.26)	187	0.97 (0.77-1.21)	0.94 (0.73-1.20)	0.95 (0.74–1.21)	285	1.52 (1.22-1.89)	1.14 (0.89-1.46)	1.15 (0.90–1.47)	
Q3	626	0.96 (0.82-1.12)	0.86 (0.73-1.02)	0.86 (0.72–1.01)	274	0.73 (0.59-0.91)	0.77 (0.61-0.98)	0.76 (0.60–0.96)	352	1.20 (0.96-1.49)	0.94 (0.74-1.20)	0.93 (0.73–1.19)	
Q4	341	1.03 (0.88-1.22)	0.97 (0.82-1.15)	0.98 (0.83–1.16)	190	0.90 (0.72-1.13)	0.91 (0.72-1.15)	0.91 (0.72–1.15)	151	1.15 (0.90-1.47)	1.02 (0.80-1.32)	1.05 (0.81–1.35)	
Q5	312	1.01 (0.86-1.20)	0.97 (0.81-1.15)	0.98 (0.82–1.17)	163	0.88 (0.70-1.11)	0.88 (0.69-1.12)	0.88 (0.69–1.13)	149	1.15 (0.90-1.47)	1.07 (0.83-1.37)	1.10 (0.85–1.42)	

Network Scores: HR (95% CI)	Total Population (n=84,729)				Males (n=30,131)				Females (n=54,598)				p for interaction
	Cases	Model 1	Model 2	Model 3	Cases	Model 1	Model 2	Model 3	Cases	Model 1	Model 2	Model 3	
Seafood: Crustacean	2,001	0.93 (0.87-0.99)	0.95 (0.89-1.02)	0.96 (0.89–1.02)	947	0.93 (0.85-1.02)	0.92 (0.83-1.01)	0.91 (0.83–1.00)	1,054	0.93 (0.85-1.02)	0.99 (0.90-1.08)	1.00 (0.91–1.10)	0.742
Q1	238	1.00 (ref)	1.00 (ref)	1.00 (ref)	121	1.00 (ref)	1.00 (ref)	1.00 (ref)	117	1.00 (ref)	1.00 (ref)	1.00 (ref)	
Q2	454	1.26 (1.08-1.48)	1.07 (0.90-1.27)	1.08 (0.90–1.28)	189	1.05 (0.83-1.32)	1.03 (0.80-1.32)	1.04 (0.81–1.33)	265	1.45 (1.16-1.81)	1.10 (0.86-1.40)	1.10 (0.86–1.40)	
Q3	621	0.97 (0.83-1.13)	0.86 (0.72-1.02)	0.85 (0.72–1.01)	276	0.78 (0.62-0.97)	0.82 (0.64-1.04)	0.81 (0.63–1.03)	345	1.15 (0.92-1.43)	0.88 (0.69-1.12)	0.87 (0.68–1.11)	
Q4	328	0.99 (0.83-1.17)	0.92 (0.77-1.10)	0.92 (0.77–1.10)	162	0.82 (0.64-1.03)	0.82 (0.64-1.04)	0.81 (0.63–1.04)	166	1.15 (0.91-1.46)	1.02 (0.80-1.31)	1.03 (0.81–1.32)	
Q5	360	0.95 (0.81-1.13)	0.88 (0.74-1.05)	0.88 (0.74–1.05)	199	0.81 (0.64-1.03)	0.82 (0.65-1.04)	0.81 (0.64–1.03)	161	1.04 (0.82-1.33)	0.91 (0.71-1.17)	0.92 (0.72–1.19)	
Seafood: Mollusks	2,001	0.86 (0.79-0.93)	0.90 (0.83-0.97)	0.90 (0.83–0.98)	947	0.92 (0.82-1.03)	0.90 (0.80-1.01)	0.89 (0.79–1.00)	1,054	0.81 (0.72-0.91)	0.91 (0.81-1.03)	0.92 (0.82–1.05)	0.055
Q1	575	1.00 (ref)	1.00 (ref)	1.00 (ref)	200	1.00 (ref)	1.00 (ref)	1.00 (ref)	375	1.00 (ref)	1.00 (ref)	1.00 (ref)	
Q2	407	0.87 (0.77-0.99)	0.90 (0.79-1.03)	0.91 (0.80–1.04)	191	1.01 (0.83-1.24)	1.00 (0.82-1.22)	1.01 (0.83–1.24)	216	0.81 (0.68-0.96)	0.86 (0.73-1.02)	0.88 (0.74–1.04)	
Q3	342	0.79 (0.69-0.91)	0.84 (0.73-0.97)	0.85 (0.74–0.98)	169	0.88 (0.71-1.08)	0.86 (0.70-1.07)	0.88 (0.71–1.08)	173	0.76 (0.63-0.91)	0.86 (0.71-1.04)	0.87 (0.72–1.05)	
Q4	341	0.84 (0.73-0.97)	0.89 (0.77-1.02)	0.90 (0.78–1.04)	196	1.03 (0.84-1.26)	0.99 (0.81-1.22)	0.99 (0.81–1.22)	145	0.72 (0.59-0.88)	0.83 (0.68-1.02)	0.85 (0.69–1.04)	
Q5	336	0.75 (0.65-0.87)	0.81 (0.70-0.93)	0.81 (0.70–0.94)	191	0.85 (0.70-1.05)	0.82 (0.67-1.01)	0.82 (0.66–1.01)	145	0.70 (0.57-0.85)	0.84 (0.68-1.03)	0.86 (0.69–1.06)	
Seafood: Processed	2,001	0.96 (0.89-1.04)	0.97 (0.90-1.05)	0.97 (0.90–1.06)	947	0.93 (0.83-1.04)	0.93 (0.83-1.04)	0.91 (0.81–1.03)	1,054	0.98 (0.88-1.09)	1.01 (0.91-1.12)	1.02 (0.91–1.13)	0.824
Q1	254	1.00 (ref)	1.00 (ref)	1.00 (ref)	118	1.00 (ref)	1.00 (ref)	1.00 (ref)	136	1.00 (ref)	1.00 (ref)	1.00 (ref)	
Q2	670	1.17 (1.01-1.36)	0.98 (0.83-1.17)	0.97 (0.82–1.15)	271	0.94 (0.75-1.17)	0.96 (0.75-1.23)	0.96 (0.75–1.22)	399	1.35 (1.10-1.65)	0.97 (0.77-1.23)	0.95 (0.75–1.20)	
Q3	360	1.05 (0.90-1.24)	0.95 (0.80-1.13)	0.95 (0.80–1.13)	184	0.98 (0.78-1.24)	1.00 (0.78-1.28)	1.00 (0.78–1.28)	176	1.08 (0.86-1.36)	0.89 (0.70-1.14)	0.89 (0.70–1.14)	
Q4	326	1.05 (0.89-1.25)	0.95 (0.80-1.13)	0.95 (0.80–1.13)	175	0.98 (0.77-1.24)	0.99 (0.77-1.26)	0.98 (0.76–1.25)	151	1.08 (0.85-1.36)	0.90 (0.70-1.15)	0.90 (0.70–1.15)	
Q5	391	1.06 (0.90-1.24)	0.96 (0.81-1.14)	0.96 (0.81–1.14)	199	0.88 (0.69-1.11)	0.89 (0.70-1.13)	0.87 (0.68–1.11)	192	1.21 (0.97-1.51)	1.01 (0.80-1.28)	1.01 (0.79–1.28)	
Seaweed	2,001	1.00 (0.90-1.12)	1.03 (0.92-1.15)	1.05 (0.94–1.19)	947	1.13 (0.95-1.33)	1.07 (0.90-1.27)	1.06 (0.89–1.27)	1,054	0.93 (0.81-1.08)	1.02 (0.88-1.18)	1.06 (0.91–1.24)	0.118
Q1	494	1.00 (ref)	1.00 (ref)	1.00 (ref)	246	1.00 (ref)	1.00 (ref)	1.00 (ref)	248	1.00 (ref)	1.00 (ref)	1.00 (ref)	
Q2	387	0.93 (0.81-1.06)	0.95 (0.83-1.09)	0.97 (0.84–1.11)	198	0.97 (0.80-1.17)	0.96 (0.79-1.16)	0.96 (0.80–1.16)	189	0.89 (0.73-1.07)	0.94 (0.77-1.13)	0.96 (0.79–1.16)	
Q3	328	0.91 (0.79-1.04)	0.94 (0.81-1.08)	0.96 (0.83–1.10)	144	0.91 (0.74-1.11)	0.88 (0.71-1.08)	0.88 (0.72–1.09)	184	0.91 (0.75-1.10)	0.98 (0.81-1.19)	1.01 (0.83–1.23)	
Q4	397	0.86 (0.75-0.98)	0.90 (0.78-1.03)	0.91 (0.80–1.05)	192	0.91 (0.75-1.10)	0.89 (0.74-1.08)	0.89 (0.73–1.09)	205	0.82 (0.68-0.98)	0.89 (0.74-1.07)	0.93 (0.76–1.12)	
Q5	395	1.01 (0.88-1.15)	1.04 (0.91-1.19)	1.07 (0.93–1.23)	167	1.13 (0.93-1.38)	1.07 (0.87-1.30)	1.05 (0.85–1.30)	228	0.93 (0.78-1.12)	1.04 (0.87-1.25)	1.10 (0.91–1.34)	

HR: Hazard ratio; CI: confidence interval
Model 1: adjusted for age and sex (for total respondents only)
Model 2: adjusted for education, city, marriage status, income group, job group, previous history of hypertension, hyperlipidemia, or diabetes, BMI, and waist circumference, added to Model 1
Model 3: adjusted for drinking status, smoking status, exercise, and total energy intake, added to Model 2
p for interaction was computed using Model 3 with interaction term of sex*food group
