# Supplementary material for: Network Approach to Evaluate the Effect of Diet on Stroke or Myocardial Infarction Using Gaussian Graphical Model
Source: Nutrients. 2025 May 8;17(10):1605. doi: 10.3390/nu17101605 (PMC12114211; doi:10.3390/nu17101605)
Supplement: Supplementary file 1 [file nutrients-17-01605-s001.zip › STROBE Checklist 20250430.pdf]

STROBE checklist of items that should be included in reports of cross-sectional case-control observational studies with detailed referencing of requirements to the text of the paper.

	Item No	Recommendation
**Title and abstract**	1	(*a*) Indicate the study's design with a commonly used term in the title or the abstract<br>Cohort study as stated in the Abstract on page 1 and Material and Methods on pages 2 and 3.
		(*b*) Provide in the abstract an informative and balanced summary of what was done and what was found<br>Provided in Abstract on page 1.
**Introduction**		
Background/rationale	2	Explain the scientific background and rationale for the investigation being reported<br>Included in the Introduction on pages 1 and 2.
Objectives	3	State specific objectives, including any prespecified hypotheses<br>Included in the Introduction on page 2.
**Methods**		
Study design	4	Present key elements of study design early in the paper<br>Included in Materials and Methods on pages 2 and 3.
Setting	5	Describe the setting, locations, and relevant dates, including periods of recruitment, exposure, follow-up, and data collection<br>Included in Materials and Methods on pages 2, 3 4.
Participants	6	(*a*) *Cohort study*—Give the eligibility criteria, and the sources and methods of selection of participants. Describe methods of follow-up<br>*Case-control study*—Give the eligibility criteria, and the sources and methods of case ascertainment and control selection. Give the rationale for the choice of cases and controls<br>*Cross-sectional study*—Give the eligibility criteria, and the sources and methods of selection of participants<br>Included in Materials and Methods on pages 2 and 3.
		(*b*) *Cohort study*—For matched studies, give matching criteria and number of exposed and unexposed<br>*Case-control study*—For matched studies, give matching criteria and the number of controls per case<br>Not applicable.
Variables	7	Clearly define all outcomes, exposures, predictors, potential confounders, and effect modifiers. Give diagnostic criteria, if applicable<br>Included in the Materials and Methods on pages 3 and 4.
Data sources/ measurement	8*	For each variable of interest, give sources of data and details of methods of assessment (measurement). Describe comparability of assessment methods if there is more than one group<br>Included in the Materials and Methods on pages 3 and 4.
Bias	9	Describe any efforts to address potential sources of bias<br>Addressed in the limitations paragraph pages 16 and 17
Study size	10	Explain how the study size was arrived at<br>Included in the Materials and Methods on page 3 (Figure 1)

Quantitative variables	11	Explain how quantitative variables were handled in the analyses. If applicable, describe which groupings were chosen and why<br>Included in the Materials and Methods on pages 4 and 7.
Statistical methods	12	(*a*) Describe all statistical methods, including those used to control for confounding<br>Included in the Materials and Methods on pages 4 and 7,8.
		(*b*) Describe any methods used to examine subgroups and interactions<br>Included in the Materials and Methods on page 8 (stratified analysis).
		(*c*) Explain how missing data were addressed<br>Included in the Materials and Methods on page 7
		(*d*) *Cohort study*—If applicable, explain how loss to follow-up was addressed<br>*Case-control study*—If applicable, explain how matching of cases and controls was addressed<br>*Cross-sectional study*—If applicable, describe analytical methods taking account of sampling strategy Included in the Materials and Methods on pages 8.
		(*e*) Describe any sensitivity analyses Not applicable.
**Results**		
Participants	13*	(a) Report numbers of individuals at each stage of study—eg numbers potentially eligible, examined for eligibility, confirmed eligible, included in the study, completing follow-up, and analysed<br>Included in the Materials and Methods on page 3 (Figure 1) and Results page 8
		(b) Give reasons for non-participation at each stage<br>Not applicable.
		(c) Consider use of a flow diagram<br>Included in the Materials and Methods on page 3 (Figure 1)
Descriptive data	14*	(a) Give characteristics of study participants (eg demographic, clinical, social) and information on exposures and potential confounders<br>Included in the Results on page 8.
		(b) Indicate number of participants with missing data for each variable of interest<br>Not reported
		(c) *Cohort study*—Summarise follow-up time (eg, average and total amount) Included in the Results on page 8.
Outcome data	15*	*Cohort study*—Report numbers of outcome events or summary measures over time
		Case-control study—Report numbers in each exposure category, or summary measures of exposure
		Cross-sectional study—Report numbers of outcome events or summary measures<br>Included in the Results on page 8, and summarized in Tables 2,3 and 4.
Main results	16	(*a*) Give unadjusted estimates and, if applicable, confounder-adjusted estimates and their precision (eg, 95% confidence interval). Make clear which confounders were adjusted for and why they were included<br>Included in the Results on pages 8,9 and summarized in Tables 2,3, and 4
		(*b*) Report category boundaries when continuous variables were categorized<br>Included in the Results on pages 8,9 and summarized in Tables 2,3, and 4
		(*c*) If relevant, consider translating estimates of relative risk into absolute risk for a meaningful time period. Not applicable.
Other analyses	17	Report other analyses done—eg analyses of subgroups and interactions, and sensitivity analyses<br>Included in the Results on pages 8,9,14 and summarized in Tables 2,3, and 4 (stratified by

		sex)
**Discussion**		
Key results	18	Summarise key results with reference to study objectives<br>Included in the Discussion on page 14.
Limitations	19	Discuss limitations of the study, taking into account sources of potential bias or imprecision. Discuss both direction and magnitude of any potential bias<br>Included in the Discussion on pages 16 and 17.
Interpretation	20	Give a cautious overall interpretation of results considering objectives, limitations, multiplicity of analyses, results from similar studies, and other relevant evidence<br>Included in the Discussion on pages 14 to 17.
Generalisability	21	Discuss the generalisability (external validity) of the study results<br>Included in the Discussion on page 16 and 17.
**Other information**		
Funding	22	Give the source of funding and the role of the funders for the present study and, if applicable, for the original study on which the present article is based<br>Provided in the text on pages 18.
